# Fully Organic Self-Powered Electronic Skin with Multifunctional and Highly Robust Sensing Capability

**DOI:** 10.34133/2021/9801832

**Published:** 2021-02-20

**Authors:** Lijuan Song, Zheng Zhang, Xiaochen Xun, Liangxu Xu, Fangfang Gao, Xuan Zhao, Zhuo Kang, Qingliang Liao, Yue Zhang

**Affiliations:** ^1^Beijing Advanced Innovation Center for Materials Genome Engineering, Beijing Key Laboratory for Advanced Energy Materials and Technologies, University of Science and Technology Beijing, Beijing 100083, China; ^2^State Key Laboratory for Advanced Metals and Materials, School of Materials Science and Engineering, University of Science and Technology Beijing, Beijing 100083, China

## Abstract

Electronic skin (e-skin) with skin-like flexibility and tactile sensation will promote the great advancements in the fields of wearable equipment. Thus, the multifunction and high robustness are two important requirements for sensing capability of the e-skin. Here, a fully organic self-powered e-skin (FOSE-skin) based on the triboelectric nanogenerator (TENG) is developed. FOSE-skin based on TENG can be fully self-healed within 10 hours after being sheared by employing the self-healing polymer as a triboelectric layer and ionic liquid with the temperature sensitivity as an electrode. FOSE-skin based on TENG has the multifunctional and highly robust sensing capability and can sense the pressure and temperature simultaneously. The sensing capability of the FOSE-skin based on TENG can be highly robust with no changes after self-healing. FOSE-skin based on TENG can be employed to detect the arm swing, the temperature change of flowing water, and the motion trajectory. This work provides a new idea for solving the issues of monofunctional and low robust sensing capability for FOSE-skin based on TENG, which can further promote the application of wearable electronics in soft robotics and bionic prosthetics.

## 1. Introduction

The human skin possesses an excellent tactile sensation to sense the pressure and temperature change [1]. Moreover, the self-healing ability of human skin enables these sensing capabilities with the restorability against mechanical damage [1]. If these capabilities can be reconstructed on the electronics such as the electronic skin (e-skin), the e-skin will be very useful in the emerging field of soft robotics and bionic prosthetics [2–6]. Accordingly, various sensing components and materials have been employed to give e-skin with sensing capability and high robustness [7–11]. The major sensing components for reported e-skin include transistors, capacitance, and resistance sensors [8, 12–14]. These sensing components usually rely on a power supply to work, which may cause the overall system bulky and greatly limit the practical utilization for e-skin. Therefore, the triboelectric-electrostatic induction effect has been proposed to construct a self-powered e-skin without power supply [15, 16]. The self-powered e-skin based on the triboelectric nanogenerator (TENG) has been employed on the human-machine interaction [17–24], wearable electronics, and medical device [25–35]. Nevertheless, the reported self-powered e-skin could only detect pressure but not temperature, which is insufficient to achieve multiple sensing for practical applications. To give the self-powered e-skin with high robustness, self-healing materials have been employed. Conventionally, self-healing materials make use of dynamic reversible intermolecular interactions including hydrogen bonds, metal-ligand coordination bonds, and dynamic covalent bonds [36–39]. Among them, the hydrogen bonds are the most widely adopted due to the advantages of directionality, moderate strength, and short healing time. The self-healing PDMS and hydrogel have been employed as a triboelectric layer and electrode, respectively, for the self-powered e-skin [40]. However, this design could not give a self-powered e-skin with a self-healing ability of the whole device, which makes the self-powered e-skin to easily fail and thus to have low robustness. Moreover, the reported self-healing and self-powered e-skin usually needs to be heated or costs a long time to achieve self-healing, which is inconvenient to the practical applications [41–48]. Hence, it is urgent to find a method to resolve the single function and low robustness of the sensing capability for the self-powered e-skin.

In this work, a fully organic self-powered e-skin (FOSE-skin) based on TENG is developed. The FOSE-skin based on TENG can achieve the multifunctional sensing of pressure and temperature with high sensitivity and fast response time. By employing a self-healing polymer as a triboelectric layer and temperature-sensitive ionic liquid as the electrode, the FOSE-skin based on TENG has a fully self-healing ability of the whole device to ensure high robustness. Thus, the sensing capability of FOSE-skin based on TENG can be restored completely without external treatment after being sheared. The developed FOSE-skin based on TENG can detect the arm swing and the temperature change of flowing water. Besides, a 3 × 3 pixel sensing multistage sensation matrix composed of the developed FOSE-skin based on TENG can perceive the motion trajectory. This FOSE-skin based on TENG shows bright prospects in many application fields including soft robotics and bionic prosthetics.

## 2. Results

**Figure 1:**
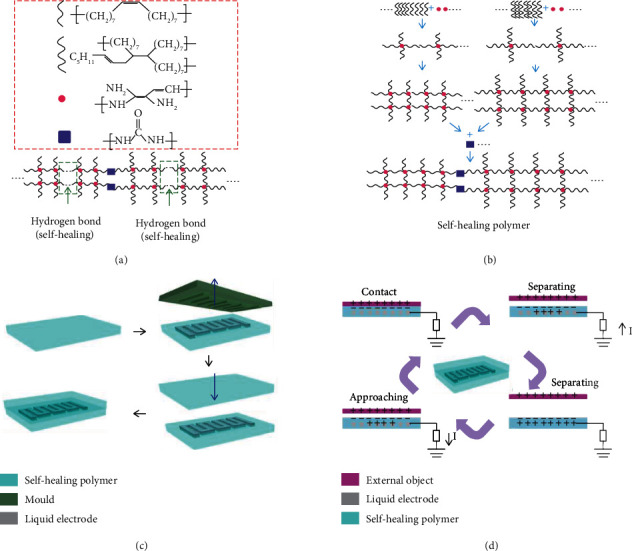
Chemical structure of the self-healing polymer and design of the FOSE-skin based on TENG: (a) the molecule and hydrogen bonds contained in the polymer; (b) the preparation process and chemical structure of the polymer; (c) schematic diagram for constructing the FOSE-skin based on TENG; (d) the pressure sensing principle of the FOSE-skin based on TENG.

Figure 1 shows the preparation of the self-healing polymer and the construction of FOSE-skin based on TENG. As shown in Figures 1(a) and 1(b), the self-healing polymer was polymerized by the two-step method. The DM-80 firstly reacted with DETA to get an oligomer, and then, the urea was added to polymerize and obtain the self-healing polymer. Afterward, the prepared polymer was molded to a 20 × 20 × 0.2 mm film to test the self-healing ability. As shown in Supplementary Figure S1, the sheared film can be self-healed in 5 min at room temperature indicating the excellent self-healing ability of the prepared polymer. To reveal the self-healing mechanism of the prepared polymer, the FTIR was carried out. Supplementary Figure S2 shows that the representative peaks of the self-healing polymer are located in 1641.32 cm^−1^, 2924.02 cm^−1^, and 3330.46 cm^−1^. These representative peaks demonstrate amounts of carbonyl, amino, and alkane groups existing in the self-healing polymer, respectively. A hydrogen bond will be formed between carbonyl and amino groups, in which amino is a proton donor and carbonyl is a proton acceptor. Thus, the polymer can be self-healing because the hydrogen bond has low bond energy and reversible dynamic characteristics [49]. Besides, the polymer film can be reconnected through the migration of the molecular chains. As shown in Supplementary Figure S3, the glass transition temperature was -6.5°C indicating that the molecule chains can easily move and the polymer film can be self-healed at room temperature. By employing the self-healing polymer and ionic liquid, the FOSE-skin based on TENG was constructed as shown in Figure 1(c). The insulated self-healing polymer acted as the triboelectric layer, and the ionic liquid [OMIm][PF_6_] with thermally sensitive resistance was employed as the electrode for the FOSE-skin based on TENG. The sensing principle of the FOSE-skin based on TENG is shown in Figure 1(d). When an external object contacts the self-healing polymer, the positive and negative charges on the surface of the polymer and object are induced to reach charge equilibrium. The generated negative triboelectric charges can remain on the polymer surface for a long time due to the insulating property. Once the external object separates from the polymer, the charge equilibrium is broken and the electrons will be induced from the liquid electrode into the ground to balance the negative charges on the polymer surface. Until the object is far away from the polymer enough, the number of negative charges on the polymer is equal to that of positive charges in the liquid electrodes. When the object approaches and contacts the polymer, the electrons will flow back from the ground to the liquid electrodes to reach charge equilibrium again. In this process, a pulse current signal is generated.

**Figure 2:**
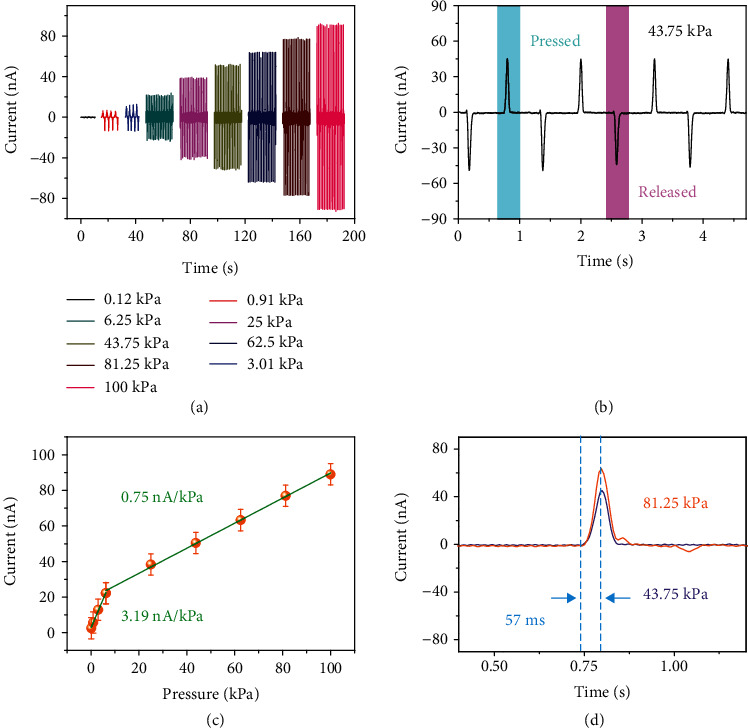
The pressure sensing capability of the FOSE-skin based on TENG: (a) the output current of the FOSE-skin based on TENG under different pressure from 0.12 kPa to 100 kPa; (b) the enlarged views of the output current at 43.75 kPa; (c) the relationship between the current and pressure; (d) the response time of the pressure sensing for the FOSE-skin based on TENG.

Figure 2 shows the pressure sensing capability of the FOSE-skin based on TENG. The output current of the FOSE-skin based on TENG under different pressure from 0.12 kPa to 100 kPa is shown in Figure 2(a). It can be seen that the output current was stably and regularly increased with the higher pressure. Moreover, the pressing process can be revealed by the current curve of 43.75 kPa for the FOSE-skin based on TENG. As shown in Figure 2(b), the blue area and pink area of the output current indicated the pressing and releasing processes, respectively. The pressure sensitivity of the FOSE-skin based on TENG is shown in Figure 2(c), which is defined as the linear relationship between the output current and pressure. At the range of 0.12~6.25 kPa pressure, the sensitivity was 3.55 nA kPa^−1^ for FOSE-skin based on TENG. While the pressure was higher than 6.25 kPa, the sensitivity of the FOSE-skin based on TENG reduced to 0.70 nA kPa^−1^. This phenomenon may be related to the well-known saturation in the triboelectric charge accumulation and contact area increasing [50]. By calculating the peak time of the output current curve, the 57 ms response time can be obtained for the FOSE-skin based on TENG, as shown in Figure 2(d). There have been researches that the range of pressure as human interacted with the outside world is about equivalent to 10 kPa, which just matches with the pressure area of high sensitivity for the developed FOSE-skin [51, 52]. Besides, the FOSE-skin has enough flexibility and conformability due to its fully organic structure design. Thus, the FOSE-skin is suitable for employing as wearable equipment on soft robotics and bionic prosthetics.

**Figure 3:**
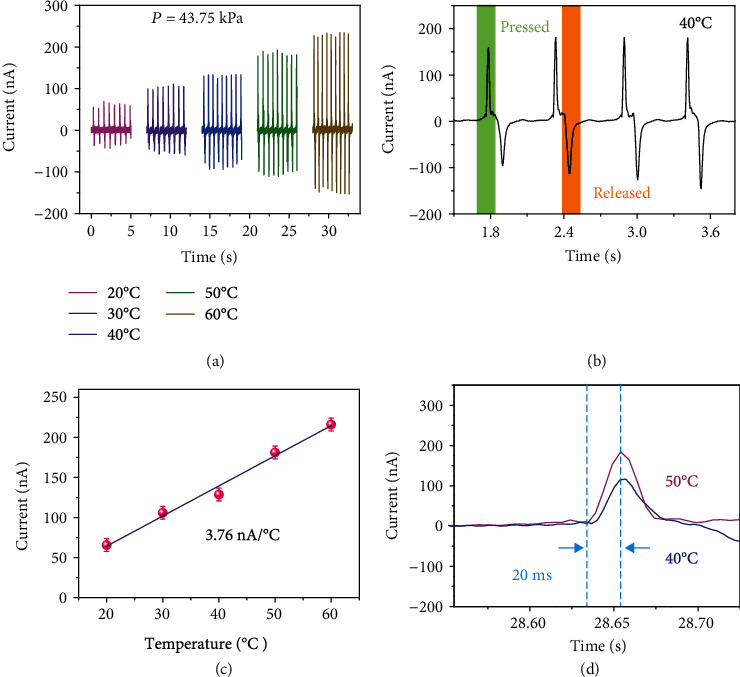
The temperature sensing capability of the FOSE-skin based on TENG: (a) the output current of the FOSE-skin based on TENG under different temperatures from 20°C to 60°C; (b) the enlarged views of the output current at 40°C; (c) the relationship between the output current and temperature; (d) the response time of the temperature sensing for the FOSE-skin based on TENG.

The FOSE-skin based on TENG can sense not only the pressure but also the temperature change. This capability is attributed to the liquid electrode which is composed of ionic liquid with temperature-sensitive resistance. As shown in Supplementary Figure S4, the resistance decreases and the conductivity increase for the ionic liquid with the temperature increasing. The change of electrode resistance leads to the different output current of the FOSE-skin based on TENG at the same pressure. As shown in Figure 3(a), the output current with the 43.75 kPa pressure was increased significantly at higher temperatures due to the decreased electrode resistance for the FOSE-skin based on TENG. The detail of the output current curve at 40°C and 43.75 kPa is shown in Figure 3(b). The green region of the output current indicated the pressing process, and the orange region of the output current represented the releasing process for the FOSE-skin based on TENG. The sensitivity of the temperature sensing for the FOSE-skin based on TENG is 3.76 nA °C^−1^, as shown in Figure 3(c). Additionally, the response time of pressure is changed for the FOSE-skin based on TENG with the temperature increasing. Figure 3(d) shows that the response time of 43.75 kPa pressure sensing was about 20 ms indicating the shorter response time of the FOSE-skin based on TENG at 40°C and 50°C.

**Figure 4:**
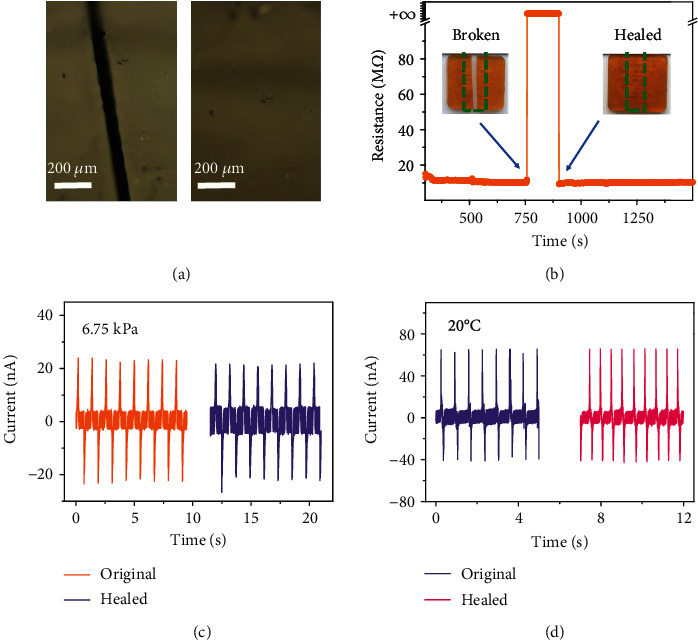
Self-healing ability of the FOSE-skin based on TENG: (a) optical microscope images of the damaged and healed FOSE-skin based on TENG at 20°C for 10 hours; (b) the self-healing process of the liquid electrode for FOSE-skin based on TENG; (c) the output current of the FOSE-skin based on TENG at 6.75 kPa and after self-healing; (d) the output current of the FOSE-skin based on TENG at 20°C and after self-healing.

Figure 4 shows that the self-healing ability of the whole device is a critical design for the FOSE-skin based on TENG with highly robust sensing capability. The self-healing ability of the FOSE-skin based on TENG was visualized by using an optical microscope as shown in Figure 4(a). The scar on the surface of the FOSE-skin based on TENG can be self-healed after 10 hours at room temperature without external treatment. For further demonstration of the self-healing ability of the whole device, the FOSE-skin based on TENG was completely cut off as shown in Supplementary Figure S5. The disconnected FOSE-skin based on TENG was put into contact for self-healing at room temperature, and 10 hours later, the FOSE-skin based on TENG can be restored to its original state. Besides, there was no decay of the electrode resistance. As shown in Figure 4(b), the electrode broke and the resistance value became infinity once the FOSE-skin based on TENG was cut off. However, the electrode resistance can be immediately restored as before when the FOSE-skin based on TENG has bonded together again. This ultrafast self-healing ability of the electrode is due to the fluidity of an ionic liquid and the capillary effect which can ensure no leak out of ionic liquid from the polymer matrix. Thus, the FOSE-skin based on TENG has a highly robust sensing capability with no changes after self-healing as shown in Figures 4(c) and 4(d). Moreover, the FOSE-skin based on TENG can work continuously for 36000 cycles which proves the excellent fatigue resistance of the FOSE-skin based on TENG as shown in Supplementary Figure S6. The developed FOSE-skin based on TENG with highly robust sensing capability will meet the applications of soft robotics and bionic prosthetics which usually requires electronics to have sufficient robustness to bear mechanical damage.

**Figure 5:**
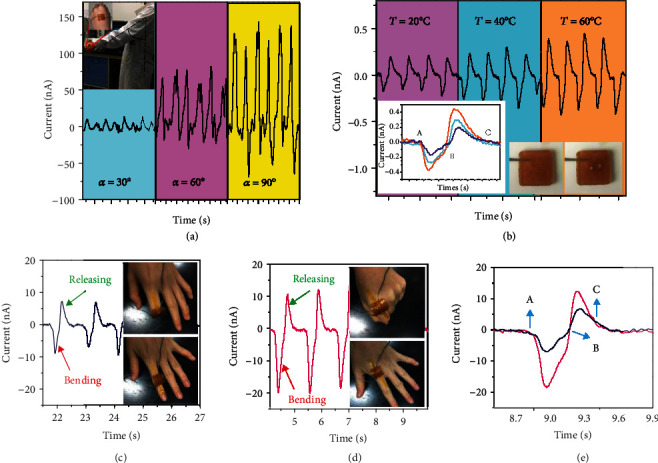
The motion and temperature sensing capability of the FOSE-skin based on TENG: (a) the detection of arm swing with different angles by the FOSE-skin based on TENG; (b) the detection of flowing water with different temperatures by the FOSE-skin based on TENG; (c) the output current of the FOSE-skin based on TENG driven by second interphalangeal joint bending and releasing; (d) the output current of the FOSE-skin based on TENG driven by metacarpophalangeal joint bending and releasing; (e) the output current signal contrast of (c) and (d).

As shown in Table S1, the developed FOSE-skin has excellent flexibility, multifunctional sensation capability, and fully self-healing ability at room temperature, which is superior to the reported self-healing and self-powered e-skin [1–9]. The developed FOSE-skin based on TENG can be used to sense the arm swing. As shown in Figure 5(a), the output current of the FOSE-skin based on TENG increased with the larger angle of the arm owing to the change in the electric field around the body. Video S1 shows the detection of arm swing with different angles by the FOSE-skin based on TENG. Besides, the FOSE-skin based on TENG can be employed to sense the temperature change of the flowing water. Figure 5(b) shows that the output current of the FOSE-skin based on TENG was increased with the higher temperature of the water. Thus, the pressure and temperature change caused by water on the surface of the FOSE-skin based on TENG can be detected simultaneously. When water drops at 60°C flow on the FOSE-skin, the temperature of the water will gradually decrease, which can result in a larger electrode resistance and lower current response for FOSE-skin. Thus, a downward trend of the current response will be demonstrated, which is more obvious as the water drops flow more slowly for the FOSE-skin. However, the common self-powered sensors based on PTFE only demonstrate constant current response as the hot water drops flow on the device. Thus, the developed FOSE-skin has a more intuitive sensing capability on the flow rate of the hot water drops. Furthermore, the process of water flowing can be obtained by the output current curve of the FOSE-skin based on TENG. As indicated by the points a–c in the inset of Figure 5(b), the water began to contact, flowed through, and then completely left the surface of the FOSE-skin based on TENG. Besides, the FOSE-skin based on TENG can be used to distinguish the motions of different joints. Figures 5(c) and 5(d) show the output current when the FOSE-skin based on TENG detects the motion of the interphalangeal joint and metacarpophalangeal joint. The motion of the metacarpophalangeal joint generates the higher output current due to the larger contact area between the metacarpophalangeal joint and FOSE-skin based on TENG. Video S2 and S3 show the detection of interphalangeal and metacarpophalangeal joint bending with different angles by the FOSE-skin based on TENG. Figure 5(e) and S7 show the detail of the output current for the joint bending and the relationship between the bending angle and the output current for the FOSE-skin. Points a and b indicate that the joint began to bend and contact with the FOSE-skin based on TENG to reach the largest angle. Moreover, point c shows that the joint separates from the FOSE-skin based on TENG and returns to the original state completely. When the joint was bending at a larger angle, the FOSE-skin will cover the joint more tightly with a larger contact area and pressure. Once the joint is returned, the FOSE-skin will separate from the joint and a larger triboelectric potential will be generated with a higher current output of e-skin during the contact-separation process.

**Figure 6:**
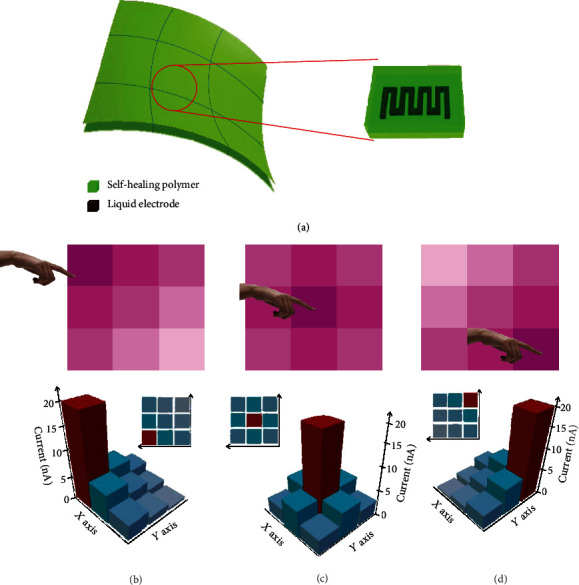
The multistage sensation matrix composed of the FOSE-skin based on TENG. (a) Schematic of the multistage sensation matrix with 3 × 3 pixels. (b–d) The top 2D intensity profile shows the pressure applied by a finger sliding on the multistage sensation matrix. The bottom 3D color mappings represent the strain distribution on the multistage sensation matrix.

Figure 6 shows the developed FOSE-skin based on TENG being applied in the multistage sensation matrix system. The FOSE-skin based on TENG with 20 mm × 20 mm × 2 mm is defined as an element. A 3 × 3 pixel sensing multistage sensation matrix system was constructed by using these elements as shown in Figure 6(a). Thus, the sensation matrix system possessed a pressure mapping capability to achieve the detection of touch position and trajectory. Figures 6(b)–6(d) show that the sensation matrix system can detect the corresponding position and area of the touch by the 2D/3D intensity mapping when a finger slides on the multistage sensation matrix. Besides, the trajectory for the slide motion can be further revealed by analyzing the generated current signal of each element in the sensation matrix system. The above device design allows the FOSE-skin based on TENG to detect the location and trajectory of the motion as human skin does.

## 3. Discussion

In summary, a FOSE-skin based on TENG is developed by the design of a self-healing polymer and has the multifunctional and highly robust sensing capability. The FOSE-skin based on TENG has multifunctional capability of sensing pressure and temperature without an external power supply owing to the triboelectric-electrostatic induction effect and thermal sensitivity mechanism. The sensitivity and response time of the pressure sensing is 0.7 nA kPa^−1^ and 57 ms, respectively, for FOSE-skin based on TENG. The temperature change can be detected by the FOSE-skin based on TENG with a 3.76 nA °C^−1^ sensitivity and 20 ms response time. Besides, the whole FOSE-skin based on TENG could be self-healed in 10 hours at room temperature upon being sheared. This self-healing ability of the whole device enables a highly robust sensing capability with no changes after self-healing for the FOSE-skin based on TENG. The developed FOSE-skin based on TENG can be employed to detect the arm swing, the temperature change of flowing water, and the motion trajectory. This work provides a new method to design a self-powered e-skin with multifunctional and robust sensing capability for applications in soft robotics and bionic prosthetics.

## 4. Materials and Methods

### 4.1. Preparation of the Self-Healing Polymer

The self-healing polymer was synthesized adopting Leibler's method. DM-80 (a mixture of 80 wt% diacid and 10 wt% triacid) 5.17 g and diethylenetriamine (DETA) 1.70 g were magnetically stirred continuously at 160°C in a nitrogen atmosphere. The resultant was then dissolved in 12.5 ml chloroform and washed with 12.5 ml deionized water and 4 ml methanol solution three times. Afterward, the residual chloroform was removed by rotary evaporation. Finally, the obtained product was polymerized with 20 wt% urea at 135°C for 24 hours at nitrogen protection.

### 4.2. Fabrication of the FOSE-Skin Based on TENG

The prepared self-healing polymer was hot-pressed at 110°C to form a 20 mm × 20 mm × 1 mm film as the substrate. The groove of 0.5 mm × 0.5 mm was printed on the substrate and was filled with an ionic liquid of 1-octyl-3-methylimidazolium hexafluorophosphate ([OMIm][PF_6_]) as an electrode. Finally, another 20 mm × 20 mm × 2 mm substrate without grooves was combined with the electrode by using chloroform to construct a FOSE-skin based on TENG.

### 4.3. Characterization and Electrical Measurement

The morphology of the polymer was investigated by field emission scanning electron microscopy (SEM, FEI Quanta 3D). The functional groups and chemical bonds in the self-healing polymer were characterized by a Fourier transform infrared (FTIR) spectrometer (Thermo Scientific, Nicolet iS50). An electrometer and a low-noise current preamplifier were used to measure the output current of the FOSE-skin based on TENG (Keithley 6517B System and Stanford SR570). The self-healing ability of the electrode for the FOSE-skin based on TENG was characterized by a multimeter (Keithley, DMM7510, 7.5-Digit).

## Data Availability

Supporting information is available free of charge via the Internet at http://pubs.acs.org.

## Supplementary Materials

Supplementary 1 Figure S1: (a) the photographs of the supramolecular self-healing polymer. (b–d) The self-healing process of the 20 mm × 20 mm × 0.2 mm polymer film. Figure S2: Fourier transform infrared spectra of the self-healing polymer films. Figure S3: the glass transition temperature of the self-healing polymer. Figure S4: measured resistance and electrical conductivity of [OMIm][PF6] at different temperatures. Figure S5: the self-healing process of the e-skin. Figure S6: the current stability of the FOSE-skin based on TENG under continuous work of 18000 s. Figure S7: the current response of the e-skin with different bending angles at the elbow location. Table S1: some recent important works about self-healing and self-powered e-skin.

Supplementary 2 Video S1: the detection of arm swing with different angles by the FOSE-skin based on TENG.

Supplementary 3 Video S2: the detection of interphalangeal joint bending with different angles by the FOSE-skin based on TENG.

Supplementary 4 Video S3: the detection of metacarpophalangeal joint bending with different angles by the FOSE-skin based on TENG.

## References

[1] (2017). Skin to e-skin. *Nature Nanotechnology*.

[2] Chen G., Matsuhisa N., Liu Z. (2018). Plasticizing silk protein for on-skin stretchable electrodes. *Advanced Materials*.

[3] Dickey M. D. (2017). Stretchable and soft electronics using liquid metals. *Advanced Materials*.

[4] Zhao S., Zhu R. (2017). Electronic skin with multifunction sensors based on thermosensation. *Advanced Materials*.

[5] Hua Q., Sun J., Liu H. (2018). Skin-inspired highly stretchable and conformable matrix networks for multifunctional sensing. *Nature Communications*.

[6] Miyamoto A., Lee S., Cooray N. F. (2017). Inflammation-free, gas-permeable, lightweight, stretchable on-skin electronics with nanomeshes. *Nature Nanotechnology*.

[7] Cooke M. E., Jones S. W., Ter Horst B. (2018). Structuring of hydrogels across multiple length scales for biomedical applications. *Advanced Materials*.

[8] He Y., Liao S., Jia H., Cao Y., Wang Z., Wang Y. (2015). A self-healing electronic sensor based on thermal-sensitive fluids. *Advanced Materials*.

[9] Wang H., Zhu B., Jiang W. (2014). A mechanically and electrically self-healing supercapacitor. *Advanced Materials*.

[10] Liu X., Su G., Guo Q. (2018). Hierarchically structured self-healing sensors with tunable positive/negative piezoresistivity. *Advanced Functional Materials*.

[11] Tee B. C., Wang C., Allen R., Bao Z. (2012). An electrically and mechanically self-healing composite with pressure- and flexion-sensitive properties for electronic skin applications. *Nature Nanotechnology*.

[12] Takei K., Takahashi T., Ho J. C. (2010). Nanowire active-matrix circuitry for low-voltage macroscale artificial skin. *Nature Materials*.

[13] Gao Y., Ota H., Schaler E. W. (2017). Wearable microfluidic diaphragm pressure sensor for health and tactile touch monitoring. *Advanced Materials*.

[14] Gao W., Ota H., Kiriya D., Takei K., Javey A. (2019). Flexible electronics toward wearable sensing. *Accounts of Chemical Research*.

[15] Ma M., Zhang Z., Liao Q. (2017). Self-powered artificial electronic skin for high-resolution pressure sensing. *Nano Energy*.

[16] Zhang Q., Liang Q., Liao Q. (2017). Service behavior of multifunctional triboelectric nanogenerators. *Advanced Materials*.

[17] Ai Y., Lou Z., Chen S. (2017). All rGO-on-PVDF-nanofibers based self-powered electronic skins. *Nano Energy*.

[18] Zang Y., Shen H., Huang D., Di C. A., Zhu D. (2017). A dual-organic-transistor-based tactile-perception system with signal-processing functionality. *Advanced Materials*.

[19] Zhang Q., Jiang T., Ho D. (2018). Transparent and self-powered multistage sensation matrix for mechanosensation application. *ACS Nano*.

[20] Lou Z., Chen S., Wang L. (2017). Ultrasensitive and ultraflexible e-skins with dual functionalities for wearable electronics. *Nano Energy*.

[21] Mu C., Song Y., Huang W. (2018). Flexible normal-tangential force sensor with opposite resistance responding for highly sensitive artificial skin. *Advanced Functional Materials*.

[22] Xiong J., Lin M. F., Wang J., Gaw S. L., Parida K., Lee P. S. (2017). Wearable all-fabric-based triboelectric generator for water energy harvesting. *Advanced Energy Materials*.

[23] Shi Q., Wang H., Wu H., Lee C. (2017). Self-powered triboelectric nanogenerator buoy ball for applications ranging from environment monitoring to water wave energy farm. *Nano Energy*.

[24] Shen J., Li Z., Yu J., Ding B. (2017). Humidity-resisting triboelectric nanogenerator for high performance biomechanical energy harvesting. *Nano Energy*.

[25] Yang W., Chen J., Wen X. (2014). Triboelectrification based motion sensor for human-machine interfacing. *ACS Applied Materials & Interfaces*.

[26] Su Y., Wang J., Wang B. (2020). Alveolus-inspired active membrane sensors for self-powered wearable chemical sensing and breath analysis. *ACS Nano*.

[27] Zhou Z., Padgett S., Cai Z. (2020). Single-layered ultra-soft washable smart textiles for all-around ballistocardiograph, respiration, and posture monitoring during sleep. *Biosensors and Bioelectronics*.

[28] Chen G., Li Y., Bick M., Chen J. (2020). Smart textiles for electricity generation. *Chemical Reviews*.

[29] Meng K., Zhao S., Zhou Y. (2020). A wireless textile-based sensor system for self-powered personalized health care. *Matter*.

[30] Zhang N., Huang F., Zhao S. (2020). Photo-rechargeable fabrics as sustainable and robust power sources for wearable bioelectronics. *Matter*.

[31] Su Y., Yang T., Zhao X. (2020). A wireless energy transmission enabled wearable active acetone biosensor for non-invasive prediabetes diagnosis. *Nano Energy*.

[32] Yan C., Gao Y., Zhao S. (2020). A linear-to-rotary hybrid nanogenerator for high-performance wearable biomechanical energy harvesting. *Nano Energy*.

[33] Jin L., Xiao X., Deng W. (2020). Manipulating relative permittivity for high-performance wearable triboelectric nanogenerators. *Nano Letters*.

[34] Zhou Z., Chen K., Li X. (2020). Sign-to-speech translation using machine-learning-assisted stretchable sensor arrays. *Nature Electronics*.

[35] Chen J., Huang Y., Zhang N. (2016). Micro-cable structured textile for simultaneously harvesting solar and mechanical energy. *Nature Energy*.

[36] Cordier P., Tournilhac F., Soulie-Ziakovic C., Leibler L. (2008). Self-healing and thermoreversible rubber from supramolecular assembly. *Nature*.

[37] Mozhdehi D., Ayala S., Cromwell O. R., Guan Z. (2014). Self-healing multiphase polymers via dynamic metal-ligand interactions. *Journal of the American Chemical Society*.

[38] Ji S., Cao W., Yu Y., Xu H. (2015). Visible-light-induced self-healing diselenide-containing polyurethane elastomer. *Advanced Materials*.

[39] Ghosh B., Urban M. W. (2009). Self-repairing oxetane-substituted chitosan polyurethane networks. *Science*.

[40] Lai Y. C., Wu H. M., Lin H. C. (2019). Entirely, intrinsically, and autonomously self-healable, highly transparent, and superstretchable triboelectric nanogenerator for personal power sources and self-powered electronic skins. *Advanced Functional Materials*.

[41] Chen Y., Pu X., Liu M. (2019). Shape-adaptive, self-healable triboelectric nanogenerator with enhanced performances by soft solid-solid contact electrification. *ACS Nano*.

[42] Sun J., Pu X., Liu M. (2018). Self-healable, stretchable, transparent triboelectric nanogenerators as soft power sources. *ACS Nano*.

[43] Xun X., Zhang Z., Zhao X. (2020). Highly robust and self-powered electronic skin based on tough conductive self-healing elastomer. *ACS Nano*.

[44] Deng J., Kuang X., Liu R. (2018). Vitrimer elastomer-based jigsaw puzzle-like healable triboelectric nanogenerator for self-powered wearable electronics. *Advanced Materials*.

[45] Parida K., Kumar V., Jiangxin W., Bhavanasi V., Bendi R., Lee P. S. (2017). Highly transparent, stretchable, and self-healing ionic-skin triboelectric nanogenerators for energy harvesting and touch applications. *Advanced Materials*.

[46] Guan Q., Dai Y., Yang Y., Bi X., Wen Z., Pan Y. (2018). Near-infrared irradiation induced remote and efficient self-healable triboelectric nanogenerator for potential implantable electronics. *Nano Energy*.

[47] Park J. H., Park K. J., Jiang T. (2017). Light-transformable and -healable triboelectric nanogenerators. *Nano Energy*.

[48] Xu W., Huang L.-B., Hao J. (2017). Fully self-healing and shape-tailorable triboelectric nanogenerators based on healable polymer and magnetic-assisted electrode. *Nano Energy*.

[49] Yang Y., Urban M. W. (2013). Self-healing polymeric materials. *Chemical Society Reviews*.

[50] Zhou Y. S., Liu Y., Zhu G. (2013). In situ quantitative study of nanoscale triboelectrification and patterning. *Nano Letters*.

[51] Gerratt A. P., Sommer N., Lacour S. P., Billard A. Stretchable capacitive tactile skin on humanoid robot fingers — First experiments and results.

[52] Mascaro S. A., Asada H. H. (2001). Photoplethysmograph fingernail sensors for measuring finger forces without haptic obstruction. *IEEE Transactions on Robotics and Automation*.

