# Supplementary material for: Fully Organic Self-Powered Electronic Skin with Multifunctional and Highly Robust Sensing Capability
Source: Research (Wash D C). 2021 Feb 20;2021:9801832. doi: 10.34133/2021/9801832 (PMC7919137; doi:10.34133/2021/9801832)
Supplement: Supplementary 1 — Figure S1: (a) the photographs of the supramolecular self-healing polymer. (b–d) The self-healing process of the 20 mm × 20 mm × 0.2 mm polymer film. Figure S2: Fourier transform infrared spectra of the self-healing polymer films. Figure S3: the glass transition temperature of the self-healing polymer. Figure S4: measured resistance and electrical conductivity of [OMIm][PF6] at different temperatures. Figure S5: the self-healing process of the e-skin. Figure S6: the current stability of the FOSE-skin based on TENG under continuous work of 18000 s. Figure S7: the current response of the e-skin with different bending angles at the elbow location. Table S1: some recent important works about self-healing and self-powered e-skin. [file 9801832.f1.docx]

**Supporting information**

**Fully Organic Self-powered Electronic Skin** **with** **Multifunctional and Highly Robust Sensing Capability**

Lijuan Song ^a,b,1^, Zheng Zhang ^a,b,1^, Xiaochen Xun^a, b^, Liangxu Xu^a, b^, Fangfang Gao^a, b^, Xuan Zhao^a, b^, Zhuo Kang^a, b^, Qingliang Liao^a, b,^ *, and Yue Zhang^a, b,^ *

^a^ Beijing Advanced Innovation Center for Materials Genome Engineering, Beijing Key Laboratory for Advanced Energy Materials and Technologies, University of Science and Technology Beijing, Beijing 100083, People’s Republic of China

^b^ State Key Laboratory for Advanced Metals and Materials, School of Materials Science and Engineering, University of Science and Technology Beijing, Beijing 100083, People’s Republic of China


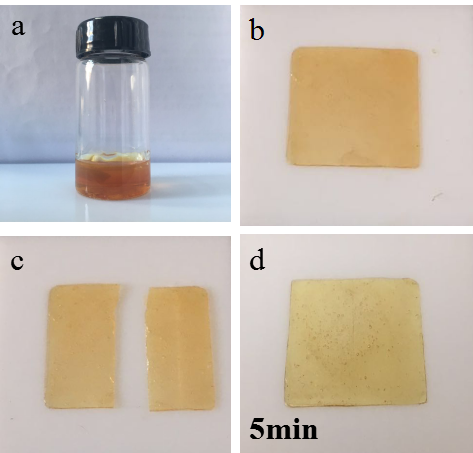


**Figure S1.** (a) The photographs of the supramolecular self-healing polymer. (b)-(d) the self-healing process of the 20mm×20mm×0.2mm polymer film.


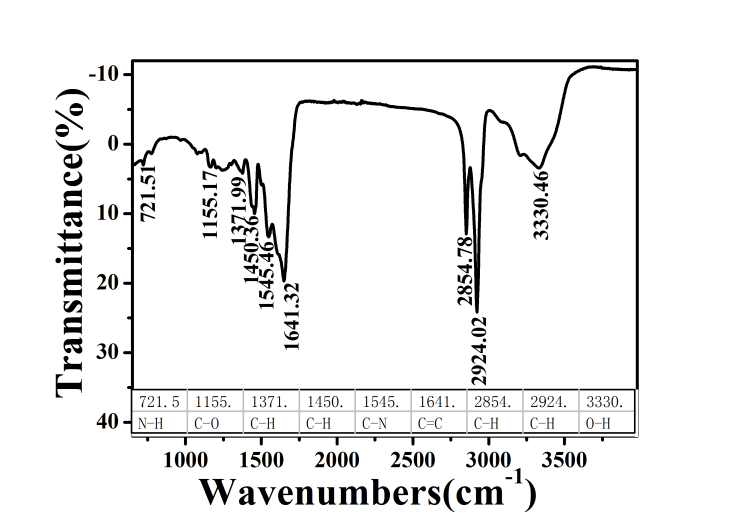


**Figure S2.** Fourier transform infrared spectra of the self-healing polymer films.

**
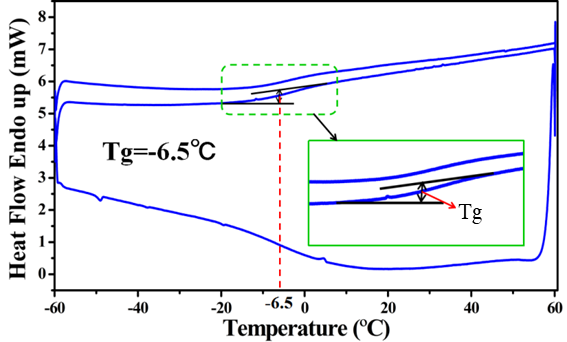
**

**Figure S3.** The glass transition temperature of the self-healing polymer.


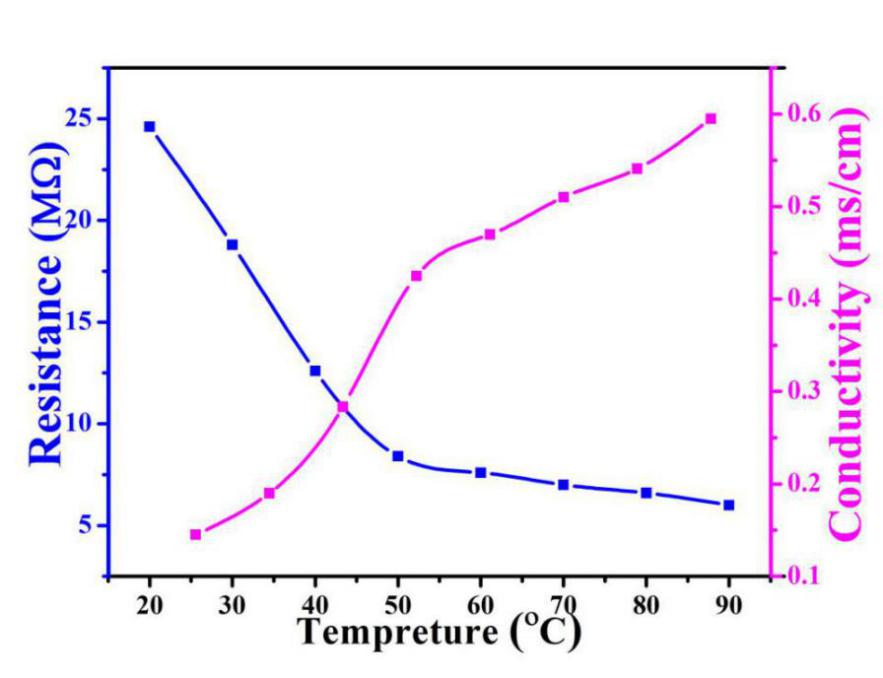


**Figure S4.** Measured resistance and electrical conductivity of [OMIm][PF6] at different temperatures.


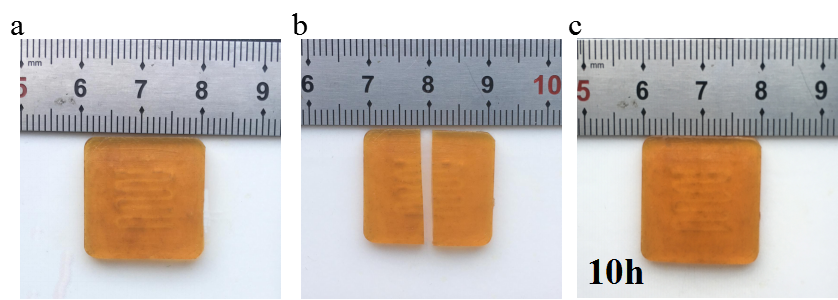


**F****igure S5.** The self-healing process of the e-skin.


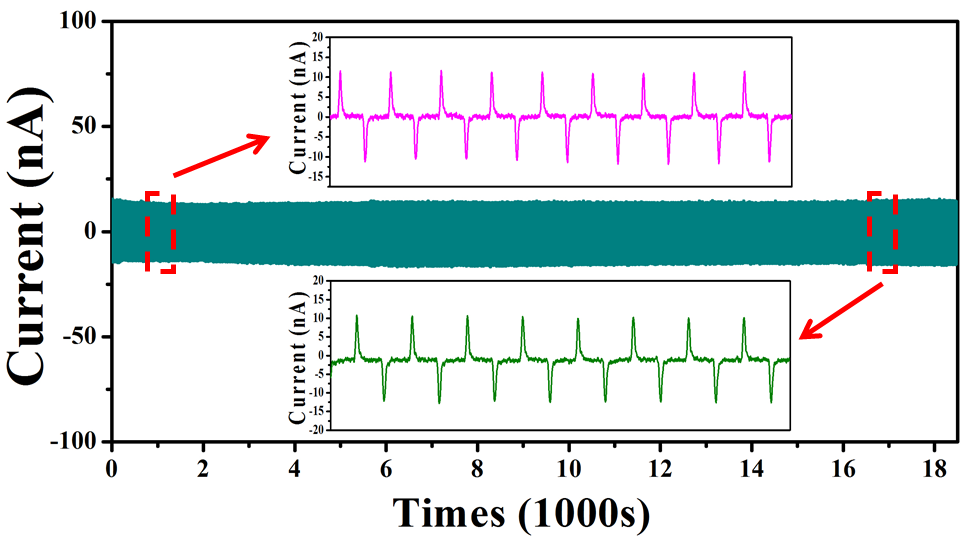


**Figure S6.** The current stability of the FOSE-skin based on TENG under continuous work of 18000 s.


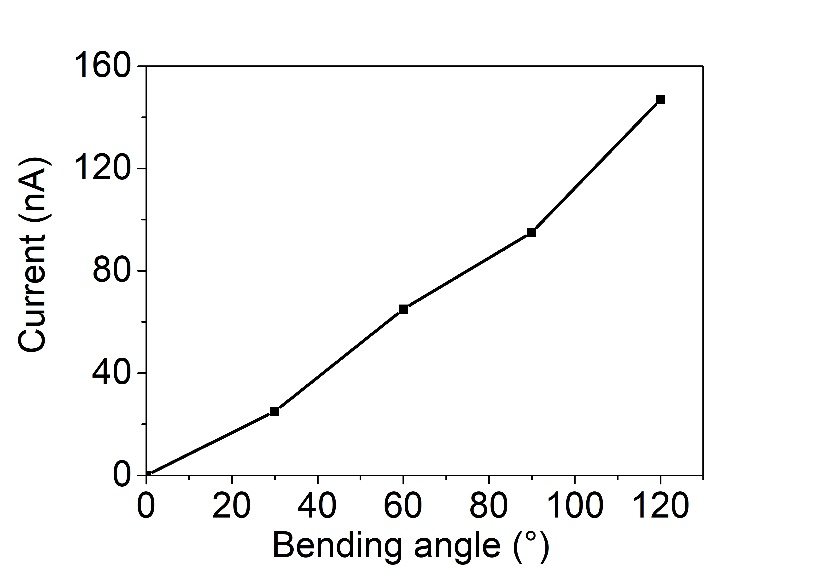


**Figure S7.** The current response of the e-skin with different bending angle at the elbow location.

Table S1. Some recent important works about self-healing and self-powered E-skin.


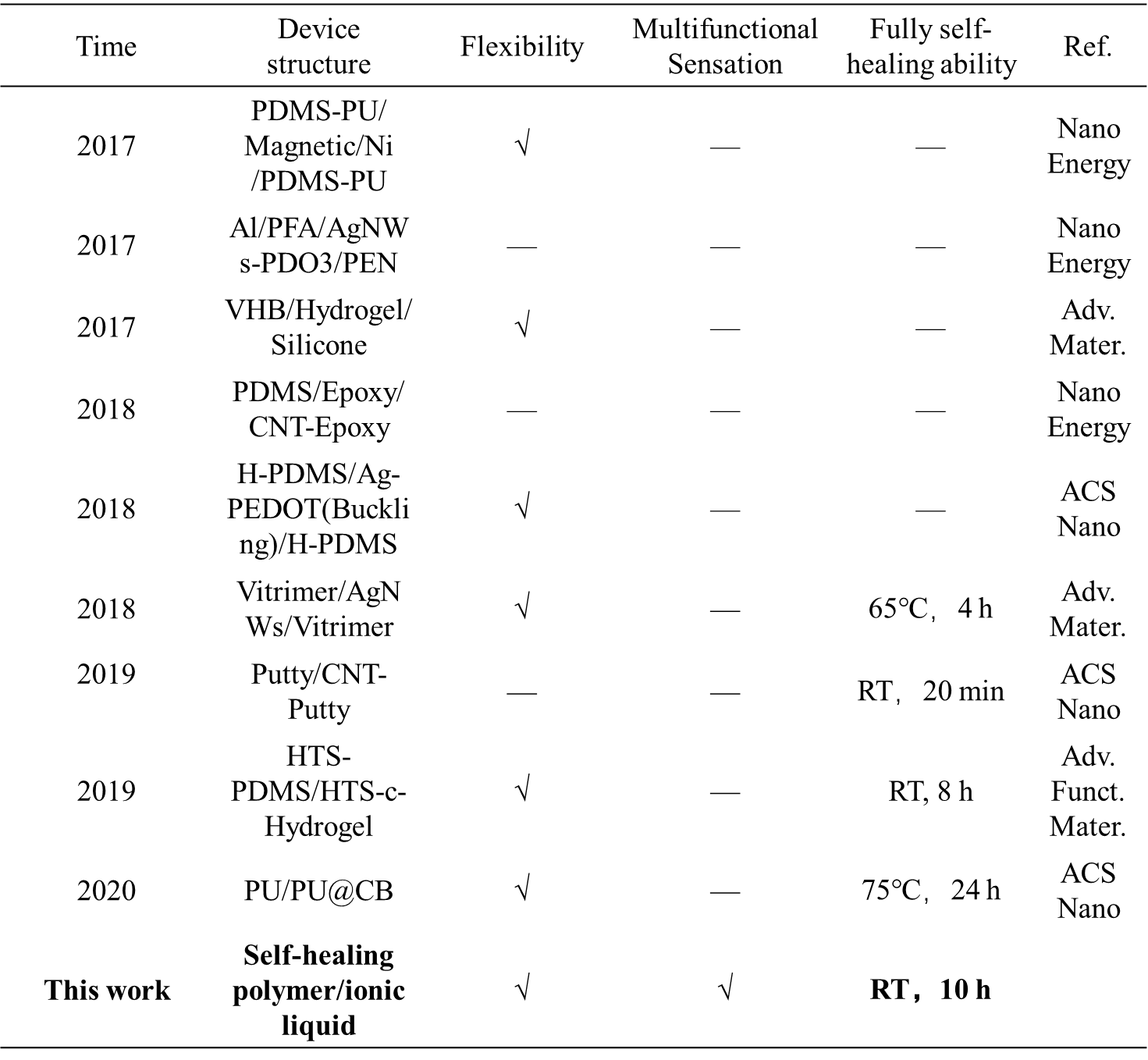


**Video S1.** The detection of arm-swing with different angle by the FOSE-skin based on TENG.

**Video S2.** The detection of interphalangeal joint bending with different angle by the FOSE-skin based on TENG.

**Video S3.** The detection of metacarpophalangeal joint bending with different angle by the FOSE-skin based on TENG.
